# Membrane Fouling Control and Treatment Performance Using Coagulation–Tubular Ceramic Membrane with Concentrate Recycling

**DOI:** 10.3390/membranes15080225

**Published:** 2025-07-27

**Authors:** Yawei Xie, Yichen Fang, Dashan Chen, Jiahang Wei, Chengyue Fan, Xiwang Zhu, Hongyuan Liu

**Affiliations:** 1 College of Civil Engineering, Zhejiang University of Technology, Hangzhou 310032, China; xyw@zjut.edu.cn (Y.X.); 221122060107@zjut.edu.cn (Y.F.); 221124060097@zjut.edu.cn (D.C.); 202005240119@zjut.edu.cn (J.W.); 211122060055@zjut.edu.cn (X.Z.); 2 Taizhou Research Institute of Intelligent Construction on Coastal Soft Soils, Zhejiang University of Technology, Hangzhou 310032, China; 3 School of Ecology and Environment, Xinyang Vocational and Technical College, Xinyang 464000, China; chengyue_fan@163.com

**Keywords:** coagulation–ultrafiltration, tubular ceramic membrane, concentrate recirculation, membrane fouling

## Abstract

A comparative study was conducted to investigate membrane fouling control and treatment performance using natural surface water as the feed source. The evaluated processes included: (1) direct filtration–tubular ceramic membrane (DF-TCM, control); (2) coagulation–tubular ceramic membrane (C-TCM); and (3) coagulation–tubular ceramic membrane with concentrate recycling (C-TCM-CR). Experimental results demonstrated that under constant flux operation at 75 L/(m^2^·h) for 8 h, the C-TCM-CR process reduced the transmembrane pressure (TMP) increase by 83% and 35% compared to DF-TCM and C-TCM, respectively. Floc size distribution analysis and cake layer characterization revealed that the C-TCM-CR process enhanced coagulation efficiency and formed high-porosity cake layers on membrane surfaces, thereby mitigating fouling development. Notably, the coagulation-assisted processes demonstrated improved organic matter removal, with 13%, 10%, and 10% enhancement in COD_Mn_, UV_254_, and medium molecular weight organics (2000–10,000 Da) removal compared to DF-TCM, along with a moderate enhancement in fluorescent substances removal efficiency. All three processes achieved over 99% turbidity removal efficiency, as the ceramic membranes demonstrate excellent filtration performance.

## 1. Introduction

Ultrafiltration (UF) is a pressure-driven membrane separation process that has attracted significant attention in the field of drinking water treatment in recent years due to its effective removal of contaminants such as suspended particles, colloids, bacteria (e.g., *E. coli* and total coliforms), and pathogenic microorganisms (e.g., *Giardia* and *Cryptosporidium*). However, its retention efficiency for small viruses (typically <20–30 nm) may be limited due to inherent membrane pore size constraints [1,2]. Currently, polymeric membranes dominate the UF membrane market, benefiting from their early development, mature fabrication processes, and lower production costs [3,4,5]. Compared to polymeric membranes, ceramic membranes (CM) demonstrate notable advantages including a long operational lifespan, high resistance to fouling, stable material properties, chemical corrosion resistance, high mechanical strength, and recyclability [6,7]. However, constrained by their higher costs, current application research on CM technology has primarily focused on industrial wastewater treatment. Its utilization in drinking water treatment remains in its infancy and warrants further exploration [6,8].

Membrane fouling remains a major challenge in CM applications, hindering its development [9,10,11]. Although fouling is inherent to filtration processes, strategies to reduce its impact must be explored to address operational issues. The mitigation of membrane fouling typically involves four principal approaches: pretreatment of feed solutions, membrane cleaning protocols, operational parameter optimization, and membrane fabrication/modification techniques [12]. Among them, pretreatment is an easy-to-use and relatively mature approach for reducing membrane fouling in practical applications [13,14].

Coagulation is a fundamental pretreatment process extensively employed in water purification systems. The primary mechanisms governing this process—including charge neutralization, sweep flocculation, and adsorption—work synergistically to destabilize colloidal particles and promote contaminant aggregation. Through these mechanisms, coagulation effectively removes a wide spectrum of pollutants, ranging from suspended particles and viral pathogens to natural organic matter [15]. By significantly lowering contaminant levels prior to UF treatment, this process mitigates membrane fouling issues [16,17]. The integrated coagulation–ultrafiltration (C-UF) process demonstrates enhanced operational advantages through membrane retention of coagulation-generated flocs: these retained flocs form a loosely structured and porous filter cake layer that adsorbs organic pollutants without substantially compromising membrane flux, thereby reducing irreversible fouling formation. Concurrently, the structural looseness of this layer facilitates efficient detachment during cleaning cycles, leading to significant mitigation of membrane fouling [17,18,19]. Compared to traditional coagulation–sedimentation–ultrafiltration processes, the C-UF process offers distinct advantages, including a smaller footprint, shorter operational procedures, simplified management, and reduced chemical consumption. These features make the C-UF process particularly suitable for small-scale rural water supply applications, where operational efficiency, resource constraints, and ease of maintenance are critical considerations [20]. Wei et al. confirmed stable high-flux operation of both C-UF and UF treating Taihu Lake water, with C-UF significantly lowering transmembrane pressure (TMP). Coagulation pretreatment enhanced UF’s limited dissolved organic removal, particularly for low molecular weight organics [21]. Zhao et al. demonstrated that coagulation with flat-sheet ceramic membranes reduced specific TMP rise and improved organics removal versus direct filtration in Yangtze River water treatment using varied coagulants [22]. By combining contaminant reduction, fouling mitigation, and operational practicality, the C-UF process presents itself as a solution for decentralized water treatment challenges.

One notable research direction in C-UF process development involves either combining this technology with complementary processes like adsorption and advanced oxidation, or designing innovative coagulants/flocculants [12]. A primary objective is to adjust floc properties during coagulation to enable the formation of a permeable cake layer with lower resistance and higher porosity on membrane surfaces [12,23]. Wang et al. demonstrated that in the C-UF process, flocs generated during the coagulation process can be reused as coagulation aids through sludge recirculation to enhance coagulation efficiency [24]. Cross-flow filtration is a common operational mode for separation membranes; the fluid generates sustained high-velocity shear forces along the membrane surface, effectively reducing membrane surface fouling while enabling continuous concentration of the feed solution [11]. Thus, in the C-UF process, cross-flow filtration can be employed to concentrate coagulated water, generating higher-density sludge recirculation that serves as recycled coagulation aids to reinforce coagulation performance.

This study proposes a comprehensive “coagulation-tubular ceramic membrane filtration” process incorporating concentrated water recirculation, capitalizing on the exceptional engineering merits of tubular ceramic membranes (TCM). By recycling residual flocs from concentrated water back to the flocculation tank, the process optimizes coagulation conditions and improves the pore structure of the filter cake layer on the TCM surface, thereby effectively delaying membrane fouling progression. Combining the concentrated water recycling mechanism of cross-flow filtration, the investigation explores the enhanced effects of residual flocs in concentrated water on coagulation processes and their regulatory mechanisms on the pore structure of membrane surface filter cake layers. This reveals the operational mechanisms underlying membrane fouling mitigation in this process, providing empirical support for CM fouling control and water purification efficiency enhancement.

## 2. Materials and Methods

### 2.1. Tubular Ceramic Membrane

TCM performance characteristics are presented in Table 1.

### 2.2. Experimental Setups

**Figure 1:**
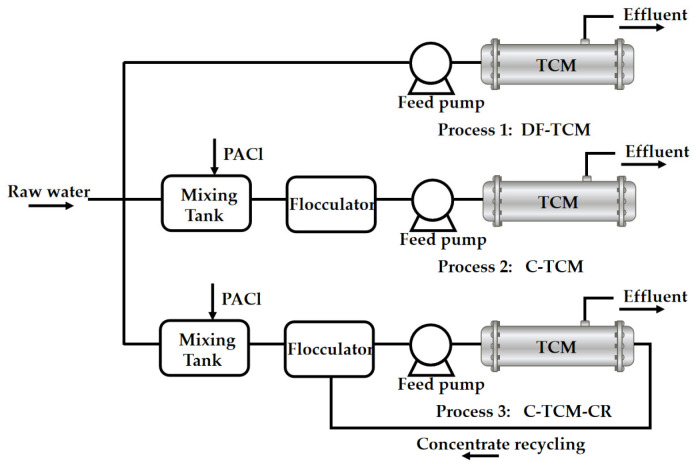
Experimental process.

This study systematically evaluated three treatment processes (see Figure 1 for the Experimental process):

The experimental steps of this study are as follows:

- Process 1: Direct filtration–tubular ceramic membrane (DF-TCM).
- Raw water was directly subjected to constant-flux dead-end filtration through TCM without pretreatment.
- Process 2: Coagulation–tubular ceramic membrane (C-TCM).
- The raw water was first mixed with polyaluminum chloride (PACl) at a dose of 1.5 mg-Al/L in a rapid mixing tank (200 rpm, 1 min), followed by flocculation in a flocculator (80 rpm, 10 min), and finally treated by constant-flux dead-end membrane filtration.
- Process 3: Coagulation–tubular ceramic membrane with concentrate recycling (C-TCM-CR).
- Based on Process 2, this modified process implemented cross-flow filtration for the TCM, operating with the cross-flow stream equivalent to 3% of the total influent flow, while ensuring complete recycling of all generated concentrate back to the flocculator.
- All three treatment processes employed identical TCM operating parameters, maintaining a constant flux of 75 L/(m^2^·h) during the 1 h filtration cycles. Each cycle concluded with an automated hydraulic backwash at 120 L/(m^2^·h) for 1 min duration to ensure optimal membrane performance.
- After every 8 filtration cycles, comprehensive chemical cleaning was conducted: Alkaline cleaning: 1000 mg/L NaClO + 1000 mg/L NaOH, injected for 1 min, circulated for 2 h; acid cleaning: 2% citric acid, injected for 1 min, circulated for 2 h.

### 2.3. Raw Water

The raw water used in this study was sourced from a municipal water treatment plant located in Zhejiang Province, China. The overall chemical characteristics of the raw water are presented in Table 2.

### 2.4. Analytical Methods

Water quality parameters, including turbidity, permanganate index (COD_Mn_), and UV_254_ absorbance, were measured according to standard methods [25,26,27,28]. Fluorescent compounds were analyzed using a fluorescence spectrophotometer (Model F-97, Shanghai Lengguang, Shanghai, China) [7,29]. Organic matter molecular weight distribution was determined by gel permeation chromatography (GPC, PL-GPC 220, Agilent Technologies, Santa Clara, CA, USA) [30]. Floc size generated during coagulation was characterized with a laser particle size analyzer (Mastersizer 3000, Malvern Panalytical, Malvern, UK) [31]. Membrane surface morphology was examined via scanning electron microscopy (SEM, Sigma 360, Carl Zeiss, Oberkochen, Germany). Total fouling (TF) of TCM was categorized into reversible fouling (RF) and irreversible fouling (IF) based on TMP variations before and after backwashing [29], calculated as:

TF = RF + IF(1)

RF = (TMP_n_ − TMP_n+1_)/TMP_0_ (2)

IF = (TMP_n+1_ − TMP_0_)/TMP_0_ (3)

where TMP_0_ is the instantaneous TMP difference measured during the initial stable operation of the TCM, (kPa); TMP_n_ and TMP_n+1_ are the instantaneous TMP difference recorded under stable operating conditions immediately after the n-th and (n + 1)-th backwashing cycle, (kPa).

## 3. Results

### 3.1. Primary Characteristics of TCM Fouling

#### 3.1.1. Evolution of TMP in TCM

**Figure 2:**
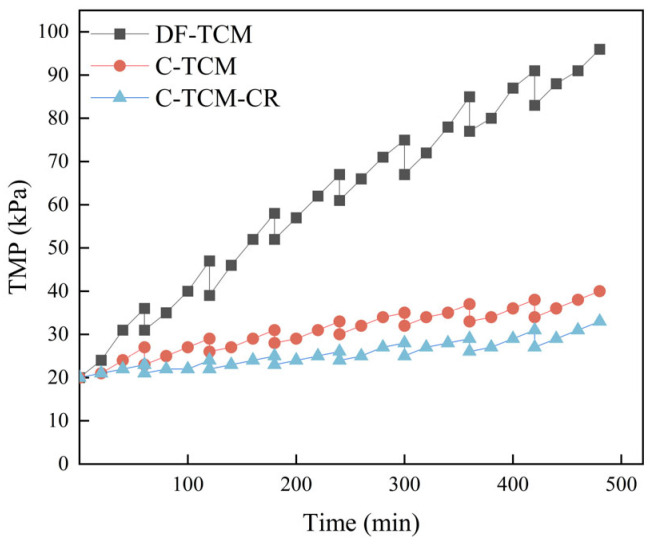
TMP variations across the three processes.

TMP serves as a vital parameter reflecting membrane fouling severity, directly correlating with hydraulic resistance during filtration [32]. Figure 2 illustrates the TMP variations across the three processes. The C-TCM-CR process demonstrated superior performance, with TMP increasing merely to 33 kPa after eight filtration cycles, representing 83% and 35% reductions in TMP elevation compared to DF-TCM and C-TCM processes, respectively. Specifically, the standalone C-TCM process reached a terminal TMP of 40 kPa, while DF-TCM exhibited the most severe fouling with a final TMP of 96 kPa. These findings conclusively indicate that: coagulation significantly alleviates membrane fouling relative to DF-TCM, and the integrated C-TCM-CR approach achieves optimal fouling mitigation through synergistic effects of enhanced coagulation and hydrodynamic control.

**Figure 3:**
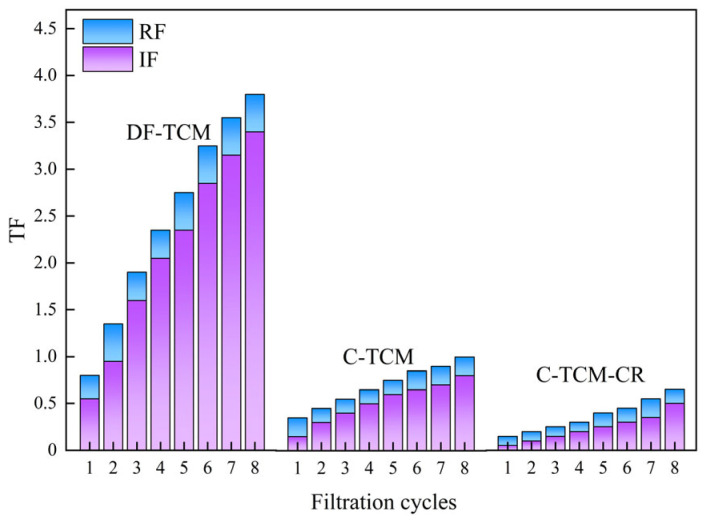
Membrane fouling characteristics variations across the three processes.

Figure 3 delineates the fouling characteristics of three operational processes across successive filtration cycles. The C-TCM-CR process demonstrated the most effective fouling control, with TF ultimately reaching 0.65 and an average RF of 0.13, which accounted for the highest proportion of TF, highlighting its superior hydraulic backwashing efficiency. This enhanced performance stemmed from synergistic mechanisms: the loose cake layer generated by coagulation mitigated fouling, cross-flow shear stress reduced cake layer adsorption load, and concentrate recirculation further optimized coagulation and floc structure. In comparison, the C-TCM process exhibited a higher TF of 1.0 at the filtration endpoint, though still significantly lower than direct filtration, with an average RF of 0.17 attributed to improved backwashing efficiency from its porous cake layer. In contrast, the DF-TCM process showed severe contamination (TF 3.8 after eight cycles) and poor reversibility, as hydraulic backwashing removed only 0.36 RF on average, reflecting limited mitigation capacity.

#### 3.1.2. Floc Size Distribution Characteristics

**Figure 4:**
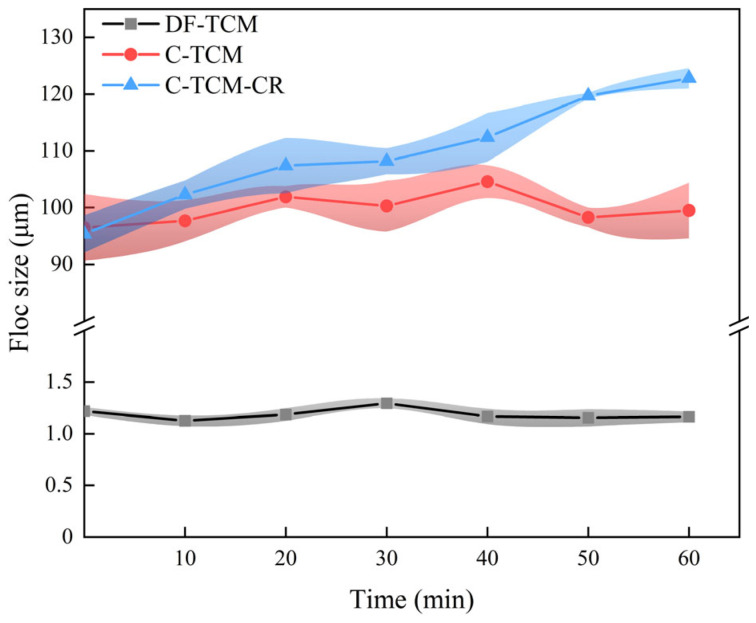
Floc size variations across the three processes.

The flocs generated during the coagulation pretreatment phase directly influence the extent of TCM fouling [12,20]. Figure 4 illustrates the variations in floc size during the flocculation stage. Experimental results revealed that the contaminant particle size in the raw water was predominantly concentrated around 0.987 μm, with a significant presence of fine particles and colloidal substances. The DF-TCM process led to the formation of dense and compact cake layers on the membrane surface, resulting in severe fouling. Coagulation facilitated the aggregation of suspended particles, colloids, and macromolecular organics into flocs with an average floc size of 99.8 μm. These flocs formed a porous cake layer on the membrane surface, effectively alleviating fouling. The C-TCM-CR process further optimized floc structure and enhanced coagulation performance. During the initial filtration phase, the average floc size was 95.4 μm. As filtration and concentrate recirculation progressed, the floc size gradually increased, reaching 122.8 μm after 1 h. In contrast, the floc size remained relatively stable in the standalone coagulation process, while direct filtration resulted in smaller particle sizes that aggravated membrane fouling. Previous studies have demonstrated that the floc size and fractal dimension in coagulation significantly influence cake layer formation. Larger flocs facilitate the development of a functional porous cake layer, which effectively intercepts smaller contaminants and mitigates membrane fouling [31,33].

#### 3.1.3. Microstructural Characterization of Cake Layers

**Figure 5:**
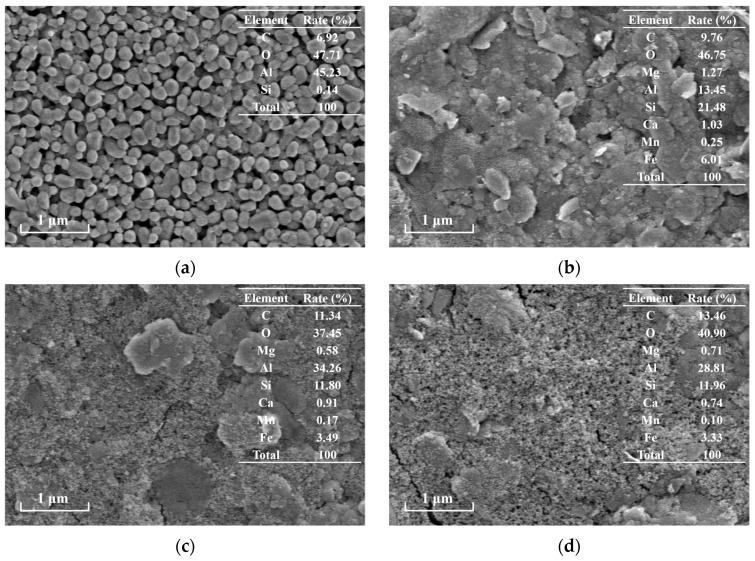
Microstructural characterization of TCM surface: (**a**) virgin membrane; (**b**) DF-TCM; (**c**) C-TCM; and (**d**) C-TCM-CR.

SEM analysis was performed to investigate the microstructural characteristics of TCM under different operational conditions, as shown in Figure 5. The virgin membrane (Figure 5a) exhibited uniformly distributed pores with well-defined morphology, and energy-dispersive X-ray spectroscopy (EDS) confirmed its primary composition of Al, O, and trace C. For operational processes, the C-TCM-CR process (Figure 5b) formed a porous cake layer with high structural integrity, whereas the standalone C-TCM (Figure 5c) showed moderately compacted deposits. In stark contrast, DF-TCM (Figure 5d) resulted in dense fouling layers completely occluding membrane pores. Elemental mapping revealed significantly elevated Al content in coagulation-assisted processes compared to DF-TCM, directly attributable to PACl residuals rather than membrane constituents. This porous architecture in C-TCM-CR was governed by hydrodynamic shear forces induced by cross-flow filtration, which dynamically maintained cake layer permeability through two synergistic mechanisms: selective removal of loosely bound foulants and continuous recirculation of concentrated particles to enhance flocs growth in the flocculator.

The combined evidence of TMP dynamics (Figure 2), floc size evolution (Figure 4), and cake layer ultrastructure (Figure 5) conclusively demonstrates that the C-TCM-CR hybrid process achieves superior fouling mitigation via three interlinked pathways: (1) optimized flocs formation enabling preliminary contaminant interception; (2) functional cake layers acting as dynamic secondary filtration barriers; and (3) enhanced hydraulic reversibility through shear-enhanced backwash efficiency. These multiscale interactions between hydrodynamic conditions, floc morphology, and membrane interface properties establish a sustainable operational paradigm for TCM applications in decentralized water treatment systems.

### 3.2. Contaminant-Specific Removal Performance

#### 3.2.1. COD_Mn_

**Figure 6:**
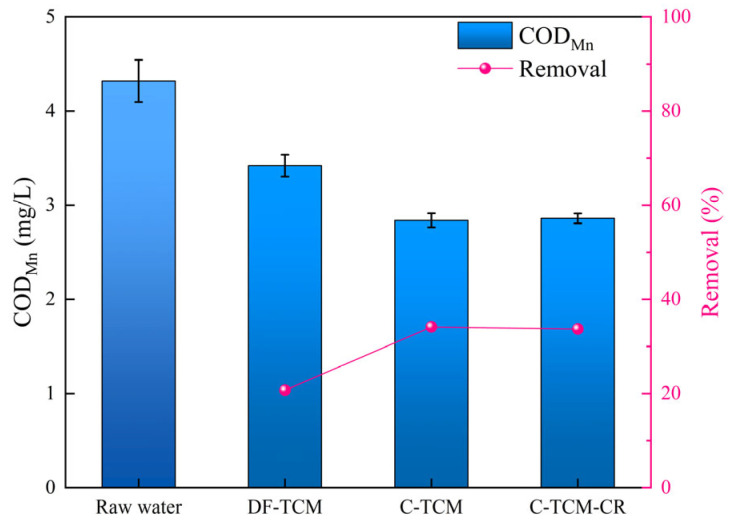
COD_Mn_ removal efficiency across the three processes.

The permanganate index (COD_Mn_), serving as a comprehensive indicator of oxidizable organic matter through potassium permanganate titration, revealed critical differences in dissolved organic matter removal across the evaluated processes. As depicted in Figure 6, the raw water exhibited an average COD_Mn_ of 4.32 mg/L. Coagulation-integrated processes demonstrated superior performance, with C-TCM and C-TCM-CR achieving 34% removal efficiency, significantly outperforming DF-TCM’s 21% reduction, which is consistent with previous studies [34,35]. This disparity stems from distinct removal mechanisms: DF-TCM primarily relies on size-exclusion (30 nm pores), exhibiting limited efficacy for sub-nanometer organics, whereas coagulation facilitates molecular aggregation of low molecular weight compounds into filterable flocs while forming porous cake layers that provide secondary adsorption sites [31,36,37].

#### 3.2.2. UV_254_

**Figure 7:**
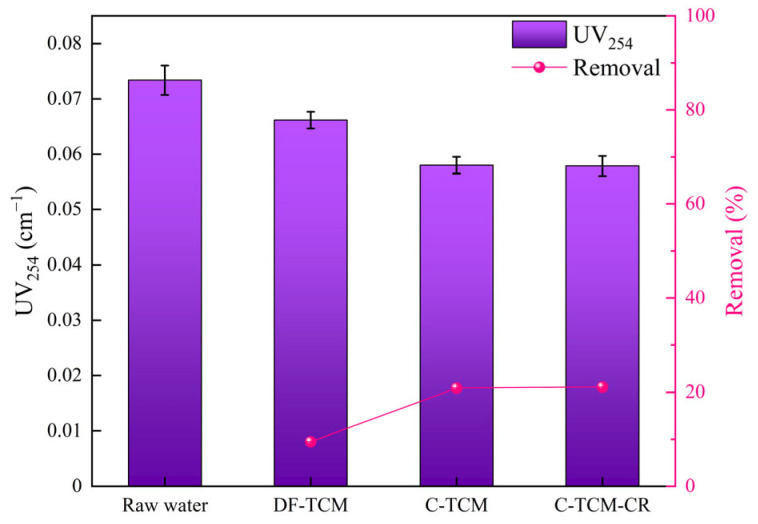
UV_254_ removal efficiency across the three processes.

UV_254_ is determined by measuring the ultraviolet light absorption of water samples at 254 nm, which reflects the concentration of aromatic compounds with conjugated double bonds and humic macromolecular organic matter [38]. Figure 7 demonstrates the UV_254_ removal efficiencies of the three processes. The raw water exhibited stable UV_254_ values ranging from 0.071 to 0.076 cm^−1^, with an average of 0.073 cm^−1^. The C-TCM and C-TCM-CR demonstrated moderate UV_254_ removal efficiency, producing an average effluent UV_254_ of 0.058 cm^−1^ and achieving a removal rate of approximately 20%. In contrast, direct filtration showed inferior performance, with an average effluent UV_254_ of 0.066 cm^−1^ and a removal rate of approximately 10%. This discrepancy is primarily attributed to the coagulation process, which promotes the aggregation of hydrophobic aromatic compounds into flocs that are more effectively retained by the ceramic membrane, thereby reducing UV_254_ [38,39].

#### 3.2.3. Turbidity

**Figure 8:**
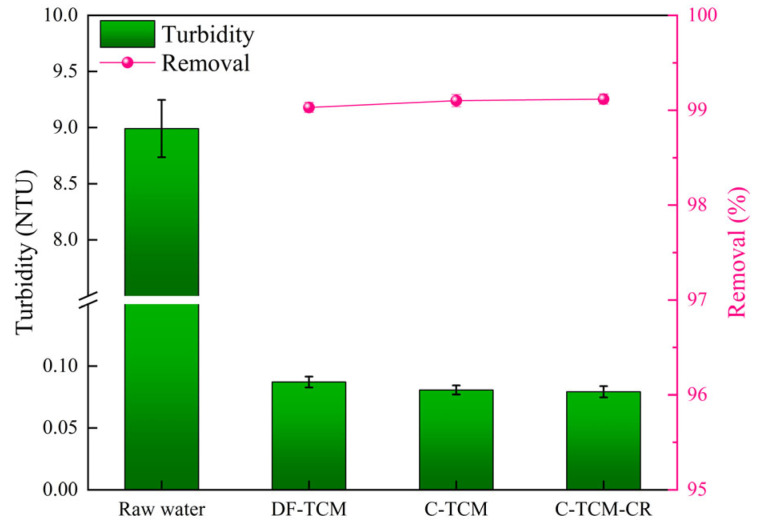
Turbidity removal efficiency across the three processes.

The raw water exhibited an average turbidity of 8.99 NTU. As illustrated in Figure 8, all three processes achieved comparable and stable turbidity removal efficiencies without statistically significant differences. Specifically, the C-TCM-CR process produced marginally lower effluent turbidity (0.079 NTU) compared to the C-TCM (0.080 NTU) and DF-TCM (0.087 NTU) processes. All three processes achieved over 99% turbidity removal efficiency, as the TCM demonstrates excellent filtration performance.

#### 3.2.4. Fluorescent Compounds

**Figure 9:**
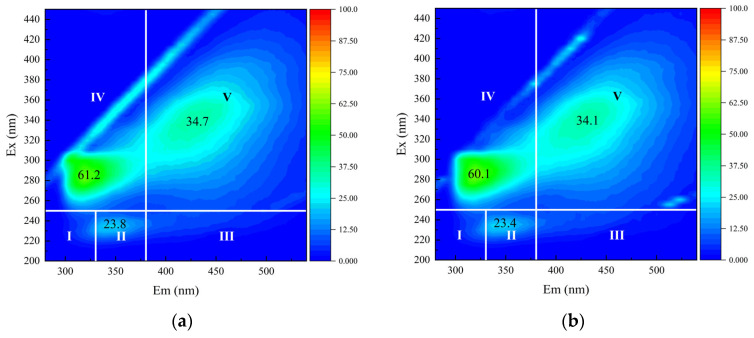

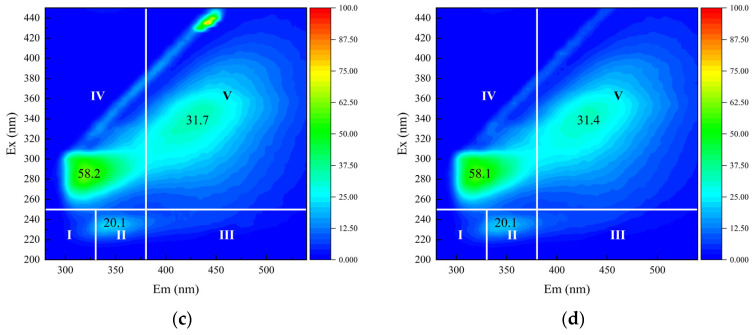
Fluorescent compounds removal efficiency across the three processes: (**a**) Raw water; (**b**) DF-TCM; (**c**) C-TCM; and (**d**) C-TCM-CR.

The fluorescence properties of humic acids, protein-like substances, and other fluorescent compounds in water were analyzed using three-dimensional excitation-emission matrix fluorescence spectroscopy, with distinct characteristic peaks corresponding to functional groups in specific fluorophores [29,40]. Figure 9 illustrates the removal efficiencies of fluorescent substances by the three treatment processes. The raw water displayed fluorescence response regions in regions II, IV, and V, attributed to aromatic proteins, soluble microbial products, and humic acids, respectively. Experimental results indicated that both C-TCM and C-TCM-CR process marginally improved the removal efficiencies of these fluorescent contaminants, achieving approximate removal rates of 5% for aromatic proteins, 9% for soluble microbial products, and 15% for humic acids. In contrast, direct filtration exhibited negligible removal efficiency. Humic acid-like pollutants were identified as a major contributor to irreversible ceramic membrane fouling. Compared to direct filtration, coagulation moderately enhanced humic acid removal, thereby alleviating membrane fouling due to their hydrophilic nature [21,31,41].

#### 3.2.5. Organic Compounds with Different Molecular Weights

**Figure 10:**
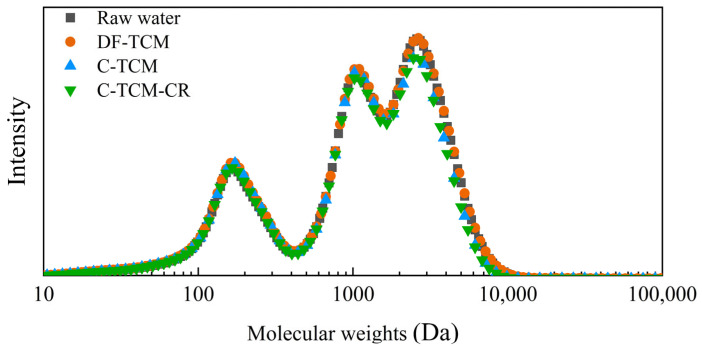
Organic compounds with different molecular weight removal efficiencies across the three processes.

GPC was employed to analyze organic matter molecular weight distribution by correlating retention times with a calibration curve established using standard molecular weight markers. The organic content was quantified based on UV absorbance at 254 nm. As shown in Figure 10, raw water primarily contained organics in the 100–10,000 Da range. TCM processes with coagulation exhibited limited removal (average 10%) of medium molecular weight organics (2000–10,000 Da), predominantly humic-like substances, consistent with findings from three-dimensional fluorescence analysis. In contrast, DF-TCM showed negligible removal of these sub-30 nm pore-sized organics. This enhanced removal in coagulation-assisted processes likely stems from porous cake layer adsorption, which prevents direct contact between low molecular weight organics (<2000 Da) and membrane surfaces, thereby reducing their permeation [42,43]. The nominal 30 nm membrane pore size corresponds to a theoretical 100,000 Da molecular weight cut-off, though this relationship is influenced by molecular shape, polarity, and other physicochemical interactions, yet actual retention efficiency for smaller organics relies heavily on synergistic cake layer filtration mechanisms rather than pure size exclusion [12,40].

## 4. Conclusions

The C-TCM-CR process achieved 83% and 35% reductions in TMP escalation, compared to DF-TCM and C-TCM during 8 h operation, respectively. This significant enhancement originated from two synergistic hydrodynamic mechanisms: (1) Cross-flow shear forces continuously disrupt foulant accumulation at the membrane surface, preferentially removing weakly adhered foulants before they undergo irreversible adhesion. This action effectively prevents progressive compaction of the cake layer while maintaining membrane permeability; (2) Simultaneously, the recycled concentrate promoted dynamic particle-fluid interactions that optimized floc architecture through enhanced collision efficiency, strengthening structural integrity. Together, these complementary mechanisms promote the formation of a highly porous cake layer with preserved flow channels, thereby substantially reducing overall hydraulic resistance. Microstructural analysis confirmed C-TCM-CR generated high-porosity cake layers with interconnected pore networks, significantly outperforming C-TCM and DF-TCM in fouling mitigation.

Coagulation pretreatment improved organic removal efficiency relative to DF-TCM, with 13%, 10%, and 10% enhancements for COD_Mn_, UV_254_, and medium molecular weight organics (2000–10,000 Da), respectively, along with a moderate increase in fluorescent substances removal. These gains stemmed from dual mechanisms: aluminum-based flocs entrapment of low molecular weight organics and limited adsorption by porous cake layers. All processes maintained >99% turbidity removal.

## Figures and Tables

**Table 1:** TCM performance characteristics.

Parameters	Specification
Material	α-Al_2_O_3_
Pore size, nm	30
Molecular weight cut-off, Da	100,000
Operating TMP, kPa	<100
Operational temperature, °C	−20–60

**Table 2:** Chemical component of the raw water in this study.

Parameters	Value
Temperature, °C	26.30–27.20
Turbidity, NTU	8.50–9.28
pH	7.47–7.88
COD_Mn_, mg/L	4.12–4.56
UV_254_, cm^−1^	0.071–0.076
Ammonia, mg/L	0.07–0.10
Fe, mg/L	0.035–0.039
Mn, mg/L	0.011–0.012
${SO}_{4}^{2-}$, mg/L	55.73–60.10
${NH}_{4}^{+}$-N	<0.02
Electrical conductivity, μS/cm	378–420
Total alkalinity, mg/L	125.3–138.8
Total hardness, mg/L	118.1–134.7

## Data Availability

The original contributions presented in this study are included in the article. Further inquiries can be directed to the corresponding author.
